# Loss of kidney function due to proteinuria, common problem with a rare cause: Answer

**DOI:** 10.1007/s00467-020-04505-7

**Published:** 2020-03-11

**Authors:** Julia Steinke, Michaela Gessner, Leonie Frauenfeld, Anna K Fischer, Wiebke Solass

**Affiliations:** 1 Institute of Pathology and Neuropathology, University Hospital Tuebingen, Eberhard-Karls University Tuebingen, Liebermeisterstr. 8, 72076 Tuebingen, Germany; 2 Department of Pediatric Nephrology, University Hospital Tuebingen, Eberhard-Karls University Tuebingen, Tuebingen, Germany

**Keywords:** Adolescent, Proteinuria, Nephrectomy, Kidney dysfunction, Lysosomal storage disease, Transplantation, Galactosialidosis

## Answer

### What is the cause of the nephrotic syndrome?

This 12-year-old girl has an autosomal recessive lysosomal storage disease called galactosialidosis. Over several years, conservative medication was maintained until, at 12 years of age, massive, uncontrollable protein loss emerged and a left-sided nephrectomy was performed which led to a controllable proteinuria for a short period of time. The counterpart kidney was also increasingly affected by the lysosomal storage disease. Interstitial accumulation of macrophages and replacement of the original kidney parenchyma lead to an insufficient renal function, presenting in this case with an uncontrollable proteinuria with massive ascites and edema. Hemodialysis and finally kidney transplantation were required to manage the situation.

## Discussion

Galactosialidosis (GS) is a rare multisystem glycoprotein storage disease caused by mutation in the *CTSA* gene. *CTSA* encodes for the lysosomal protein cathepsin A (PPCA) which protects the two enzymes b-galactosidase and neuramindase1 (NEU1) [1]. All three together form a complex, and only within this complex does b-GAL/NEU1 reach their full activity and stability. Therefore, genetic alteration and loss of function of PPCA results in a combined b-GAL/NEU1 deficiency. The clinical symptoms are secondary to accumulation of sialyl-oligosaccharides in multiple organs as a result of failed degradation. The prevalence of GS is currently unknown and is considered an orphan autosomal recessive disease, which is subdivided into three clinical subtypes according to age of onset and symptom severity.

### Early infantile type

The early infantile type is characterized by an early onset between birth and 3 months of age, and can include non-immune hydrops fetalis or even fetal loss. Symptoms can involve several organ systems, like the kidney (renal failure, proteinuria, edema), heart (cardiomegaly), eyes (corneal clouding, fundal changes), and other typical changes like skeletal changes and coarse feces. Psychomotor development is characterized by severe delay and later regression [2].

### Late infantile type

The late infantile type presents within the first 2 years of life and slowly progresses into adulthood. It is characterized by mild to absent cognitive retardation. As in the early infantile type, different organ systems can be involved: heart (valve insufficiency), growth retardation, muscular atrophy, kidney and pulmonary complications, and rarely neurological symptoms [3].

### Juvenile/adult type

The juvenile/adult type compromises the majority of GS patients. These patients are mostly of Japanese origin, and the clinical course shows a large variability. As with their early and late infantile counterparts, these cases show common features like coarse feces, skeletal changes, and ocular changes. They often suffer from cerebellar ataxia, generalized seizures, myoclonus, and a progressive cognitive impairment with mental retardation [4].

### Pathology of disease

Alterations in the *CTSA* gene lead to GS, and in the literature, different genetic alterations (deletions, insertions, missense mutations, splicing variants, etc.) have been described. To date, around 36 pathognomonic mutations have been reported [5]. It has been shown that the severity and type of symptoms may vary depending on homozygosity or compound heterozygosity [6].

### Histological changes

Histopathological examination is usually performed via renal biopsy, and nephrectomy is rare. In the present case, the renal parenchyma presented with massive, interstitial accumulation of histiocytic macrophages with clear, fine coarse cytoplasm. The foamy macrophages are preferentially located at the periphery of the organ; the renal medulla is not involved. Additionally, there is a focal interstitial lymphocytic chronic infiltrate which is intermingled with the macrophages. The renal tubules are dilated with accumulation of macrophages also in the epithelial cells, with enlarged eosinophilic cytoplasm, focal necrosis, and atrophy. Glomeruli are enlarged, plump with transformation into foamy macrophages accompanied by fibrosis. All vessels—small and middle-sized—are without histological changes. Symptomatic renal involvement has been described in a few cases with galactosialidosis [7, 8], but to our knowledge, a symptomatic nephrectomy so far has not been described in the literature.

### Therapy

Therapeutic options for GS patients are symptomatic. New therapy options have been investigated in mouse models since the 1990s, but so far, no therapeutic agent has been established. For example, Zhou et al. performed transplantation from transgenic mice with bone marrow over-expressing PPCA and showed that this leads to a complete reversal of the pathological changes in all organs, and increased enzymatic activity [9].

Enzyme replacement therapy with recombinant human PPCA has also been undertaken in mice. After 2 weeks of administration, normalized cathepsin A activity with reduction of lysosomal storage was achieved in many organ systems [10].

Another promising approach is the use of recombinant adeno-associated viral vectors expressing human PPCA, especially for the late infantile group of patients with no nephrotic symptoms [11].

### Regarding this case

Our case presented an oligosymptomatic course of a late infantile form of galactosialidosis with mainly renal involvement, which was, however, severe. To our knowledge, only one case of kidney transplantation in a patient with GS has been described previously [3], but without symptomatic nephrectomy. In our case, kidney transplantation was performed 2 years after primary nephrectomy. Both cases describe GS of the juvenile type in which renal failure with massive proteinuria and resulting nephrotic syndrome was the leading symptom.

## Conclusion

In summary, galactosialidosis is a rare autosomal recessive multisystemic congenital glycoprotein storage disease, which affects multiple organs due to accumulation of oligosaccharides. In this case, the severe involvement of the kidneys led to an uncontrollable proteinuria with chronic kidney failure. One-sided nephrectomy and adaptation of medication could not help to control the clinical features, and hemodialysis was performed until transplantation was possible.
